# The impact of perceived organizational justice on job satisfaction among police officers: a moderated mediation model involving organizational identification and supervisor loyalty

**DOI:** 10.3389/fpubh.2026.1900275

**Published:** 2026-08-06

**Authors:** Yang Zhang, YiFei Li, Wenhao Guo, Yingfan Gou, Shiwei Ren, Yali Feng, Jinyu Wang, Sheng Li

**Affiliations:** 1 School of Public Health, Gansu University of Traditional Chinese Medicine, Lanzhou, Gansu, China; 2 First Clinical Medical College of Lanzhou University, Lanzhou, Gansu, China; 3 Tailai Prison, Qiqihar, Heilongjiang, China; 4 Nursing Department, The No.2 People's Hospital Of Lanzhou, Lanzhou, Gansu, China; 5 Emergency Department, The Second Hospital of Lanzhou University, Lanzhou, Gansu, China; 6 School of Public Health, Lanzhou University, Lanzhou, Gansu, China; 7 Department of Public Health, The No.2 People's Hospital of Lanzhou, Lanzhou, Gansu, China

**Keywords:** job satisfaction, organizational identification, perceived organizational justice, police, supervisor loyalty

## Abstract

**Objective:**

To explore the mechanism through which perceived organizational justice influences police job satisfaction, and to test the mediating role of organizational identification and the moderating role of supervisor loyalty.

**Methods:**

Using stratified cluster sampling, 4,271 active-duty police officers were selected from public security agencies in City R between March and May 2026 as the study sample. The police officers were surveyed using the Organizational justice Scale, Supervisor Loyalty Scale, Job Satisfaction Scale, and Organizational Identification Scale. Statistical analysis of the collected data was performed using SPSS 27.0, PROCESS, and the Bootstrap method.

**Results:**

There were significant positive correlations (*P* < 0.01) between all variables—perceived organizational justice, organizational identification, supervisor loyalty, and job satisfaction—in pairs. Mediation analysis revealed that organizational identification partially mediated the relationship between perceived organizational justice and job satisfaction (95% CI did not include 0). The interaction term between perceived organizational justice and supervisor loyalty significantly influenced organizational identification (*P* < 0.001), with a moderated mediation index of −0.003 (95% CI did not include 0), indicating that the moderated mediation effect was established.

**Conclusion:**

Perceived organizational justice directly and positively predicts job satisfaction among police officers; organizational identification partially mediates the relationship between perceived organizational justice and job satisfaction; and supervisor loyalty negatively moderates the effect of perceived organizational justice on organizational identification. The mechanism through which perceived organizational justice influences job satisfaction among police officers is a moderated mediation effect.

## 1 Introduction

Police officers are vital guardians of the rule of law and public safety. Their physical and mental wellbeing, as well as their job satisfaction, are critical to the efficiency of the police force and social stability. Job satisfaction (1) refers to a pleasant or positive emotional state that arises when an individual evaluates their career or workplace experience, reflecting their overall attitude toward work and emotional experiences. For a long time, the police profession has been known for its high intensity, high risk, and high demands, and this group faces immense occupational stress (2). Research indicates that police officers constitute a high-stress occupational group, with a significantly higher incidence of mental health issues compared to the general population (3). Their overall mental health status is cause for concern, and job satisfaction is generally low (4). However, empirical research on job satisfaction among Chinese police officers remains scarce (5). Especially how perceived organizational justice influences job satisfaction through psychological pathways, and under what conditions this relationship may change, remains to be further explored. Therefore, exploring the key factors influencing police job satisfaction and their mechanisms of action—particularly the interaction between organizational-level factors and individual psychological factors—holds significant theoretical and practical value for enhancing police force effectiveness and maintaining organizational stability.

Among the many organizational factors that influence job satisfaction, perceived organizational justice (6)—that is, employees' subjective perceptions of justice in the allocation of organizational resources and decision-making processes—is considered a key variable. According to the fairness heuristic theory (7), when individuals face uncertainty, they rely on perceived justice as a basis for judgment in order to quickly determine whether they should trust the organization and its authority. Police work is characterized by a high degree of uncertainty and danger (2), making perceived justice particularly important in shaping police attitudes. Existing research indicates that perceived organizational justice has a significant positive impact on police job satisfaction (8). A study by Lambert et al. (9) on Chinese police officers found that procedural fairness and distributive fairness were both significantly and positively correlated with police job satisfaction. However, how perceived organizational justice influences job satisfaction, and whether there are other influencing factors in its mechanism, remains to be further investigated.

To explain this process, this study introduces organizational identification as a mediating variable. Organizational identification (10) refers to the alignment of values between an individual and an organization; it is a psychological state in which an individual defines their self-identity as a member of the organization, reflecting the individual's sense of belonging to the organization and the degree to which they have internalized its values. According to social exchange theory, when employees perceive fair treatment from the organization, they develop a sense of obligation to reciprocate. This positive interaction strengthens their psychological attachment and identification with the organization, thereby enhancing job satisfaction (11). Empirical research (12) indicates that organizational identification is positively correlated with employees' work attitudes and serves as a crucial psychological link between organizational environmental factors and individual work attitudes. Therefore, this study hypothesizes that organizational identification mediates the relationship between perceived organizational justice and job satisfaction.

Furthermore, the impact of perceived organizational justice may be moderated by individual emotional factors. In police units characterized by a highly hierarchical structure and an emphasis on obedience, immediate supervisors play a crucial role. Supervisors are not only enforcers of organizational policies but also the primary source of emotional support and resources for their subordinates. Supervisory loyalty (13) refers to a subordinate's psychological identification with their supervisor, endorsement of the supervisor's values, and display of dependent behavior—including a willingness to prioritize the supervisor's interests over their own and to proactively contribute to the supervisor's success. A survey of police officers in Liaoning Province found that supervisor commitment is a significant predictor of job satisfaction (14). A study by Barao et al. (15) on United State police officers similarly found that procedural fairness from immediate supervisors was significantly and positively correlated with officers' work motivation and their perception of supervisory legitimacy, further indicating that supervisory factors play a central role in shaping police attitudes. When police officers exhibit high loyalty toward their immediate supervisors, the immediate support and emotional recognition provided by supervisors may partially compensate for deficiencies at the organizational institutional level, thereby weakening the influence of perceived organizational justice on organizational identification. In other words, supervisor loyalty may act as a moderating factor, negatively moderating the relationship between perceived organizational justice and organizational identification. It is worth noting that although prior studies have examined supervisor-related factors (e.g., supervisor commitment, supervisor justice) as direct predictors or mediators of police work attitudes (14, 15), few have investigated supervisor loyalty as a moderator, and its negative moderating effect on the “perceived organizational justice → organizational identification” pathway has not yet been revealed.

**Figure 1:**
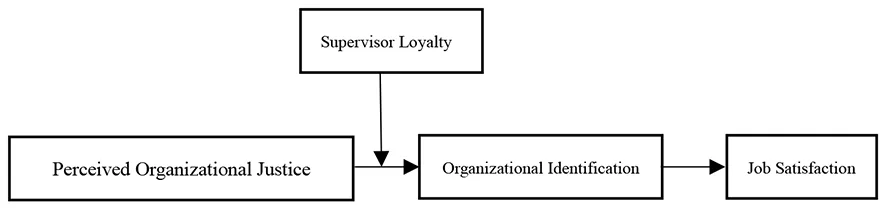
Theoretical model of police job satisfaction with moderated mediation.

Based on the above theoretical analysis, this study further developed a moderated mediation model (as shown in Figure 1). This model not only tests the mediating role of organizational identification between perceived organizational justice and job satisfaction among police officers but also introduces supervisor loyalty as a moderator to thoroughly analyze its moderating effect on the first half of the mediation path: “perceived organizational justice → organizational identification → job satisfaction.” The research findings will provide empirical evidence for enhancing job satisfaction and mental health among police officers and offer guidance for public security agencies to optimize their human resource management practices.

## 2 Materials and methods

### 2.1 Survey participants

From March to May 2026, a stratified cluster sampling method was employed. The administrative divisions of City R served as the basis for stratification, with the sample divided into two levels: the Municipal Public Security Bureau and the public security bureaus of each district (county). Within each level, cluster sampling was conducted by police specialty, and a survey was conducted of all police officers in the selected units who met the inclusion criteria. Inclusion criteria: (1) Active-duty police officers with formal civil service status; (2) Having worked in R City's public security organs for 1 year or longer; (3) Informed consent. Exclusion criteria: (1) Long-term sick leave or off-duty for training, and not on duty during the survey period; (2) Currently undergoing treatment for work-related injuries or serious illnesses. A total of 4,280 questionnaires were distributed, with 4,271 valid responses collected, resulting in a valid response rate of 99.78%. Among the respondents, 3,409 were male (79.8%) and 862 were female (20.2%); 3,519 were married or cohabiting (82.4%), while 752 were unmarried, divorced, separated, or widowed (17.6%); 645 were criminal investigators (15.1%), 894 were public security officers (20.9%), 635 were traffic police (14.9%), 110 were household registration officers (2.6%), 452 were community police (10.6%), and 1,535 were from other police units (35.9%).

### 2.2 Survey tools

#### 2.2.1 Organizational justice scale

The Organizational Perceived Justice Scale developed by Colquitt (16) was used. The scale consists of 20 items divided into four dimensions: distributive justice, procedural justice, interpersonal justice, and informational justice. A 7-point Likert scale was used, ranging from 1 (“Strongly Disagree”) to 7 (“Strongly Agree”). The mean scores for each dimension and the total score were calculated; higher scores indicate a higher level of organizational perceived justice. In this study, the Cronbach's α coefficient for this scale was 0.977. This scale has been demonstrated to possess sound reliability and validity in the Chinese cultural context (17).

#### 2.2.2 Loyalty to supervisor scale

The Supervisor Loyalty Scale developed by Chen (13) was used. The scale consists of 17 items and is divided into five dimensions: dedication, effort, following, identification with the supervisor, and internalization of the supervisor's values. A 7-point Likert scale was used, ranging from 1 (“strongly disagree”) to 7 (“strongly agree”). The mean scores for each dimension and the total score were calculated; higher scores indicate greater loyalty to the immediate supervisor. In this study, the Cronbach's α coefficient for this scale was 0.958. This scale has been shown to have adequate reliability and validity (18).

#### 2.2.3 Minnesota satisfaction questionnaire short form

The Short Minnesota Satisfaction Scale was used (19). The scale consists of 20 items divided into two dimensions: intrinsic satisfaction and extrinsic satisfaction. A 5-point Likert scale was employed, ranging from 1 (“very dissatisfied”) to 5 (“very satisfied”). The mean score was calculated, with higher scores indicating greater job satisfaction. In this study, the Cronbach's α coefficient for this scale was 0.941. The Chinese version of the Minnesota Satisfaction Questionnaire has been validated as having good reliability and validity (20).

#### 2.2.4 Organizational identification scale

The Organizational Identification Scale developed by Mael and Ashforth (21) was used. The scale consists of six items and employs a 5-point Likert scale ranging from 1 (“strongly disagree”) to 5 (“strongly agree”). The mean score was calculated, with higher scores indicating stronger organizational identification. In this study, the Cronbach's α coefficient for this scale was 0.896. This scale has been confirmed to have sound reliability and validity in the Chinese context (22).

### 2.3 Statistical analysis

Data analysis was performed using SPSS 27.0. Categorical data were described using frequencies and percentages. The Harman one-way test was used to assess common method bias across the four scales: perceived organizational justice, organizational identification, supervisor loyalty, and job satisfaction. Pearson correlation analysis was used to examine the relationships among the variables. The PROCESS plugin was used: model 4 tested the mediating effect of organizational identification between perceived organizational justice and job satisfaction, while Model 7 tested the moderating effect of supervisor loyalty in a moderated mediation model. The analysis controlled for gender, marital status, and police specialty. The significance of the mediating effect was assessed by calculating the 95% confidence interval using the Bootstrap method (5,000 repeated samples). The significance level was set at α = 0.05.

## 3 Result

### 3.1 Test for deviation from the common method

To examine the potential impact of common method bias, an analysis was conducted using Harman's one-factor test. The results showed that there were a total of eight factors with eigenvalues greater than 1. The first principal component obtained prior to rotation accounted for 18.404% of the total factor loadings, which did not exceed the critical threshold of 40%. This indicates that no serious issues of common method bias were found in the data results of this study.

### 3.2 Correlation analysis

The results of the analysis indicate that perceived organizational justice is positively correlated with job satisfaction, organizational identification, and supervisor loyalty. Job satisfaction is positively correlated with organizational identification, supervisor loyalty, and perceived organizational justice (Table 1).

**Table 1:** Correlation matrix for the variables.

Variable	1	2	3	4
Perceived organizational justice	1			
Organizational identification	0.394^**^	1		
Supervisor loyalty	0.650^**^	0.418^**^	1	
Job satisfaction	0.705^**^	0.380^**^	0.534^**^	1

Label: ^**^P < 0.01.

### 3.3 Mediating effect

**Figure 2:**
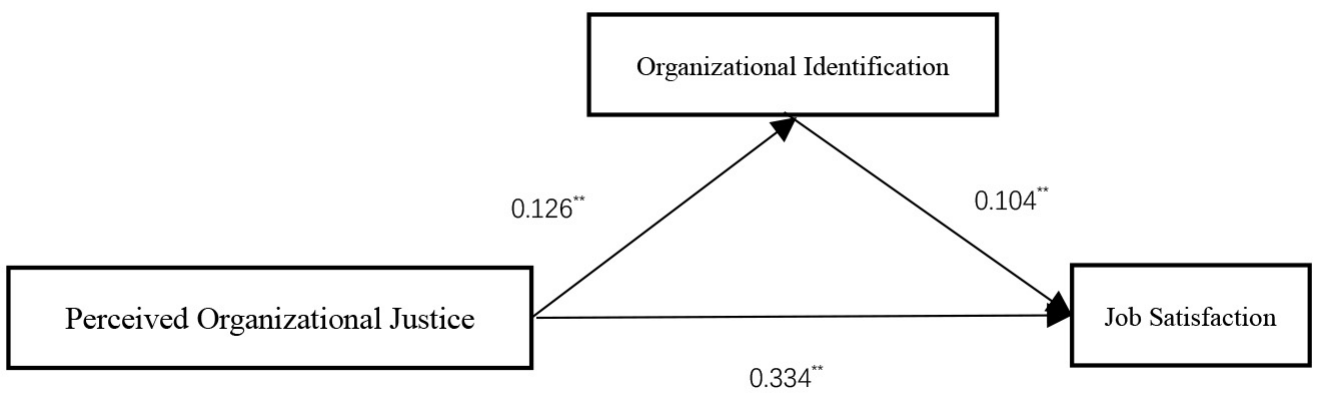
Mediating path model; Label: **P < 0.01.

Model 4, using the PROCESS package, tested the mediating effect of organizational identification between perceived organizational justice and job satisfaction. The analysis controlled for gender, marital status, and police specialty. The results showed that the total effect of perceived organizational justice on job satisfaction was significant (β = 0.359, *t* = 64.020, *P* < 0.001). After including the mediating variable organizational identification, the direct effect remained significant (β = 0.334, *t* = 55.518, *P* < 0.001). The indirect effect of organizational identification was 0.025, with a 95% CI of 0.019–0.031, which does not include zero. The results indicate that organizational identification partially mediates the relationship between perceived organizational justice and job satisfaction (Table 2). The specific path coefficients for the mediating effect are shown in Figure 2.

**Table 2:** An analysis of the effects of perceived organizational justice on job satisfaction among police officers.

Effect type	β	SE	95%CI
Total effect	0.359^**^	0.006	0.348–0.370
Direct effect	0.334^**^	0.006	0.322–0.346
Indirect effect	0.025	0.003	0.019–0.031

Label: ^**^P < 0.01.

### 3.4 Moderation effect test

We used the Process Model 7 to test the moderating effect of supervisor loyalty on the relationship between perceived organizational justice and organizational identification. As shown in Table 3, both perceived organizational justice and supervisor loyalty positively predicted organizational identification (*B* = 0.126, 0.186; both *P* < 0.001). The interaction term between perceived organizational justice and supervisor loyalty significantly predicted organizational identification (*B* = −0.027, *t* = −4.860, *P* < 0.001), with a 95% CI of −0.039 to −0.016 that does not include zero, indicating that supervisor loyalty negatively moderates the relationship between perceived organizational justice and organizational identification (Table 3).

**Table 3:** Moderated effects of perceived organizational justice on job satisfaction among police officers.

Dependent variable	Independent variable	β	SE	95%CI
Organizational identification	Perceived organizational justice	0.126^**^	0.011	0.104–0.148
	Supervisor loyalty	0.186^**^	0.012	0.162–0.210
	Perceived organizational justice × supervisor loyalty	−0.027^**^	0.006	−0.039 to −0.016
Job satisfaction	Perceived organizational justice	0.334^**^	0.006	0.322–0.346
	Organizational identification	0.104^**^	0.010	0.084–0.123

Label: ^**^P < 0.01.

We further analyzed the indirect effect of perceived organizational justice on job satisfaction when the moderator variable, supervisor loyalty, took on different levels. The results showed that for different levels of supervisor loyalty, the 95% CIs for the indirect effect were 0.012–0.021, 0.010–0.017, and 0.007–0.014, respectively, none of which included 0, indicating that the indirect effect was significant and that its effect size decreased as the level of supervisor loyalty increased. The direct effect of perceived organizational justice on job satisfaction remained constant across different levels (*B* = 0.334, 95% CI: 0.322–0.346), which is equal to the direct effect in Table 3, further confirming that supervisor loyalty negatively moderates the effect of perceived organizational justice on organizational identification. The moderated mediation index is −0.003 (95% CI: −0.005 to −0.001), which does not include 0, indicating that the moderated mediation effect holds (Table 4).

**Table 4:** An analysis of the condition effects on supervisor loyalty at different levels.

Type of effect	Supervisor loyalty	β	SE	95%CI
Indirect effect	M−1SD	0.016	0.002	0.012–0.021
	M	0.013	0.002	0.010–0.017
	M+1SD	0.010	0.002	0.007–0.014
Direct effect		0.334^**^	0.006	0.322–0.346

Label: ^**^P < 0.01; M and SD represent the mean and standard deviation of the indirect effects estimated using Bootstrap resampling.

### 3.5 Simple slope analysis

**Figure 3:**
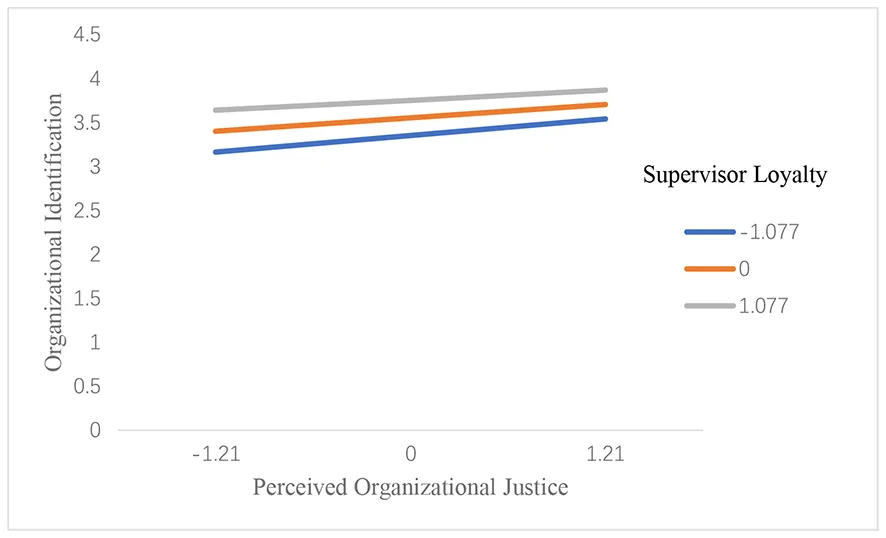
Simple slope analysis of the moderating effect of supervisor loyalty on the relationship between organizational identification and perceived organizational justice.

The results of the simple slope analysis show that when supervisor loyalty is at low and moderate levels, respectively (simple slopes = 0.156 and 0.126, respectively; both *P* < 0.001), perceived organizational justice can positively predict organizational identification; when supervisor loyalty is at high levels, perceived organizational justice still positively predicts organizational identification (simple slope = 0.097, *P* < 0.001), but the predictive effect is significantly weakened (see Figure 3). This indicates that as the level of supervisor loyalty among police officers increases, the positive simple effect of perceived organizational justice on organizational identification diminishes accordingly (Figure 3).

## 4 Discussion

### 4.1 The direct effect of perceived justice in police organizations on job satisfaction

This study found that perceived organizational justice has a positive predictive effect on police job satisfaction (β = 0.359, *P* < 0.001); that is, the higher the level of organizational justice perceived by police officers, the higher their job satisfaction, consistent with the findings of previous studies (8, 23). According to the fairness heuristic theory, the high uncertainty of police work makes officers more reliant on perceptions of justice to assess organizational trustworthiness (7, 24). When officers perceive fair treatment in areas such as distribution, procedures, interpersonal interactions, and information, it creates a psychological cue that the organization is trustworthy, thereby enhancing job satisfaction. A study (25) found that work resources (including perceived justice) positively predict police officers' wellbeing while negatively predicting emotional exhaustion, indicating that a sense of organizational justice is a crucial resource for maintaining police officers' mental health and enhancing job satisfaction. Building on this, a study (26) found that positive emotions mediate the relationship between police officers' social media use and job satisfaction, suggesting that positive psychological states directly translate into job satisfaction. Conversely, when police officers perceive unfairness, they experience negative emotions such as anger and disappointment, which in turn reduce their job satisfaction. Research by Mojallal et al. (27) confirms that experiential avoidance indirectly affects police officers' burnout, wellbeing, and productivity through increased negative emotions and reduced positive emotions, indicating that the rise in negative emotions and the decline in positive emotions collectively undermine police officers' occupational mental health and job performance. Therefore, public security agencies should prioritize fostering a sense of organizational justice by ensuring fairness and transparency in promotions, rewards, and training, establishing transparent decision-making procedures, and providing police officers with opportunities to participate in decision-making, thereby enhancing their job satisfaction.

### 4.2 An analysis of the mediating effect of organizational identification

The results of the mediation analysis indicate that police organizational identification partially mediates the relationship between perceived organizational justice and job satisfaction (β = 0.025, 95% CI: 0.019–0.031), suggesting that perceived organizational justice not only directly influences police job satisfaction but also exerts an indirect effect by enhancing their organizational identification. Social exchange theory (11) provides a theoretical explanation for this finding: when individuals perceive positive treatment within an organization, they develop a sense of obligation to reciprocate, a relationship built on trust and long-term interaction. A study by Sun et al. (28) on the Nigerian police also found that perceived organizational fairness indirectly influences police officers' organizational commitment through organizational trust and job satisfaction, further supporting the theoretical framework that perceived organizational fairness influences police officers' work attitudes through intermediate psychological mechanisms. As a motivational resource, perceived organizational justice can foster police officers' trust in the organization, leading them to internalize the organization's goals and values as part of their own beliefs, thereby enhancing job satisfaction. Han and Lee's study (29) of South Korean police also found that organizational justice (particularly distributive justice and interactional justice) positively influences police officers' positive work attitudes through the mediating effect of trust in superiors, indicating that the pathway through which a sense of organizational justice influences police officers' positive work attitudes via intermediate psychological mechanisms also holds true for police populations in East Asia. Wolfe et al. (8) found that the relationship between organizational justice and job satisfaction is moderated by uncertainty. Specifically, police officers facing higher levels of career uncertainty rely more heavily on fair treatment from supervisors to assess their job satisfaction, as fair treatment signals that the organization cares about members' interests and will provide future support. This finding directly supports the present study: when police officers perceive fair treatment in areas such as distribution, procedures, interpersonal interactions, and information, this sense of justice reduces the psychological threat posed by career uncertainty, strengthens their trust in and identification with the organization, and consequently enhances job satisfaction. In other words, a sense of organizational justice serves not only as a direct motivator but also as a signal of psychological safety, helping police officers cope with career challenges by reinforcing their organizational identification. This suggests that managers should, on the one hand, enhance the sense of organizational justice by refining institutional designs to ensure justice and transparency in promotions, rewards, and training. On the other hand, they should focus on cultivating police officers' organizational identification by conducting organizational cultural activities, promoting organizational values, and enhancing their sense of professional pride, thereby strengthening their psychological sense of belonging to the organization and fully leveraging the role of organizational identification as a vital psychological resource. It is worth noting that the mediating effect of organizational identification (indirect effect = 0.025, 95% CI: 0.019–0.031) accounts for approximately 7% of the total effect (β = 0.359). In empirical research, relatively small mediation effects are actually quite common. Walters (30) pointed out that factors contributing to small mediation effect sizes include: loss of information when variables are measured across different time points, the inclusion of control variables in the analysis, neglect of measurement error in the variables, and the use of multiple mediators. In the present study, the relatively small partial mediation effect may be attributed to the following reasons: perceived organizational justice primarily influences job satisfaction through the direct path (β = 0.334), leaving limited explanatory room for the indirect path; additionally, this study controlled for multiple covariates including gender, marital status, and police specialty, which, while enhancing the precision of the model, may have also reduced the proportion of variance that the mediating effect could explain. Furthermore, partial mediation is the expected norm in mediation analysis—causal mechanisms in the real world are typically complex, and a simple single-mediator model can hardly capture all mediating pathways. Therefore, despite the relatively small effect size, the Bootstrap 95% confidence interval (0.019–0.031) does not contain zero, still supporting the conclusion that organizational identification plays a partial mediating role between perceived organizational justice and job satisfaction. A deeper analysis reveals that organizational identification partially mediates the effect of perceived organizational justice on police job satisfaction, constituting an incomplete mediation model. This suggests that other influencing factors may exist within the indirect pathway through which perceived organizational justice affects police job satisfaction. Further exploration is needed to refine research on the relationship between perceived organizational justice and police job satisfaction.

### 4.3 The moderating effect of supervisor loyalty

To explore the mechanisms underlying job satisfaction among police officers in greater depth, this study introduced supervisor loyalty as a moderating variable into the mediation model. The results indicate that the interaction term between perceived organizational justice and supervisor loyalty significantly predicts organizational identification (B = −0.027, *t* = −4.860, *P* < 0.001), with supervisor loyalty negatively moderating the effect of perceived organizational justice on organizational identification. According to social exchange theory (11), the relationship between an organization and its employees is not merely an economic contract; more importantly, it is a socio-emotional exchange based on trust, respect, and long-term reciprocity. When a sense of organizational justice fails to meet police officers' expectations in a timely manner, high loyalty to their immediate supervisor can serve as a substitute: as representatives of the organization, supervisors can provide police officers with immediate, concrete emotional support and additional resources, thereby compensating to some extent for deficiencies in organizational-level systems.

A simple slope analysis revealed that when supervisor loyalty is low, the effect of perceived organizational justice on organizational identification is stronger (simple slope = 0.156); as supervisor loyalty increases, this effect gradually weakens (simple slopes = 0.126 and 0.097), showing a clear decreasing trend. This indicates that the positive predictive effect of perceived organizational justice on organizational identification is negatively moderated by supervisor loyalty; that is, the higher the level of supervisor loyalty, the weaker the positive effect of perceived organizational justice on organizational identification. This finding is consistent with the conclusions of Jeon and Jeong's study (31) on South Korean police officers. The study found that trust in superiors plays a negative moderating role between perceived fairness in performance evaluations and defensive silence among police officers; that is, the higher the level of trust in superiors, the stronger the inhibitory effect of perceived fairness on negative behavior. Although the dependent variables differ, both studies collectively indicate that trust in/loyalty to superiors plays a key buffering role in shaping police attitudes and behavior, moderating the impact of perceived organizational fairness on police psychology and behavior. The above model supports the validity of the moderated mediation path. From a social exchange perspective, it can be observed that when police officers perceive the same level of organizational justice, those with higher loyalty to their supervisors derive their organizational identification more from psychological attachment to their direct supervisors rather than from perceptions of justice in organizational systems. In other words, supervisor loyalty largely substitutes for the role of perceived organizational justice. Conversely, police officers with lower levels of supervisor loyalty rely more on the justice of organizational systems for their organizational identification. When perceived organizational justice is insufficient, the organizational identification of this group of officers declines significantly. Therefore, managers in public security agencies should prioritize building relationships between supervisors and subordinates, leveraging the unique role of supervisors in conveying organizational values and fostering police morale. Lambert et al. (32) found that interpersonal justice, procedural justice, and distributive justice are positively correlated with police officers' organizational commitment, indicating that a sense of organizational justice plays a positive predictive role in officers' attitudes toward the organization. This also confirms the significant influence of supervisors on police job satisfaction, suggesting that managers can enhance the organization's overall cohesion and sense of belonging by optimizing supervisors' management styles. For officers with low supervisor loyalty, particular attention should be paid to their perceptions of organizational justice, addressing any potential lack of identification through institutional improvements and enhanced communication. For officers with high supervisor loyalty, supervisors' exemplary roles can be fully leveraged. Managers can use the findings of this survey as a reference to develop differentiated management strategies tailored to officers with varying levels of supervisor loyalty, thereby enhancing their job satisfaction.

In summary, based on social exchange theory and fairness heuristic theory, this study examined the mechanism through which perceived organizational justice influences police job satisfaction and tested this moderated mediation model. It also suggested the possibility of other new mediating variables, providing a reference for future research. The main contributions of this study are twofold. First, it identifies organizational identification as a key psychological bridge through which perceived organizational justice translates into job satisfaction, thereby adding empirical evidence for the mediating pathway of “perceived organizational justice → organizational identification → job satisfaction” in a Chinese police sample. Second, and more importantly, this study reveals the negative moderating effect of supervisor loyalty on this pathway—that is, when police officers hold high loyalty toward their immediate supervisors, the positive effect of perceived organizational justice on organizational identification is weakened, suggesting that supervisor loyalty “substitutes” for the role of perceived organizational justice to some extent. This finding of a “substitution effect” differs from previous studies that primarily treated supervisor-related factors as antecedents or mediators of job satisfaction (14) instead, it positions supervisor loyalty as a moderator with a negative direction of effect—a finding that has received limited attention in the existing literature. In other words, in the highly disciplined context of police organizations, supervisor loyalty does not necessarily strengthen the effect of perceived organizational justice, but rather may weaken it, revealing a unique mechanism of supervisor-subordinate relationships in this specific context. However, this study is a cross-sectional survey, and all data were derived from self-reports by police officers. Although the Harman one-factor test—which showed that the first principal component explained 18.404% of the variance—indicates that there are no serious common-method bias issues, cross-sectional data cannot capture the dynamic changes in the relationships among perceived organizational justice, organizational identification, and job satisfaction. The mediating model proposed in this study (perceived organizational justice → organizational identification → job satisfaction) and the moderating (supervisor loyalty mediating the effect of perceived organizational justice on organizational identification) both imply temporal relationships among the variables. However, since cross-sectional data measure all variables at a single point in time, they cannot rigorously test the evolutionary sequence of these psychological processes, nor can they rule out the possibility of reverse causality (e.g., police officers with high organizational identification may be more inclined to perceive organizational justice). Therefore, the conclusions of this study should be understood as statistical associations and theoretical inferences based on cross-sectional data, rather than strict causal evidence. Future research could adopt a longitudinal design, measuring perceptions of organizational justice, the formation of organizational identification, and changes in job satisfaction at different time points to more accurately test the dynamic causal relationships among the variables; simultaneously, multi-source data (such as authentic peer evaluations and supervisor evaluations) could be introduced to further validate the stability of the supervisor loyalty moderation effect.

## Data Availability

The raw data supporting the conclusions of this article will be made available by the authors, without undue reservation.
